# New Challenges in Ocular Drug Delivery

**DOI:** 10.3390/pharmaceutics16060794

**Published:** 2024-06-11

**Authors:** Rosario Pignatello, Hugo Almeida, Debora Santonocito, Carmelo Puglia

**Affiliations:** 1 Laboratory of Drug Delivery Technology, Department of Drug and Health Sciences, University of Catania, Viale A. Doria 6, 95125 Catania, Italy; debora.santonocito@unict.it (D.S.); carmelo.puglia@unict.it (C.P.); 2 NANOMED—Research Centre for Nanomedicine and Pharmaceutical Nanotechnology, Department of Drug and Health Sciences, University of Catania, 95125 Catania, Italy; 3 UCIBIO (Research Unit on Applied Molecular Biosciences), REQUIMTE (Rede de Química e Tecnologia), MEDTECH (Medicines and Healthcare Products), Laboratory of Pharmaceutical Technology, Department of Drug Sciences, Faculty of Pharmacy, University of Porto, 4050-313 Porto, Portugal; hperas5@hotmail.com; 4 Associate Laboratory i4HB-Institute for Health and Bioeconomy, Faculty of Pharmacy, University of Porto, 4050-313 Porto, Portugal; 5 Mesosystem Investigação & Investimentos by Spinpark, Barco, 4805-017 Guimarães, Portugal

The clinical treatment of diseases affecting the eye globe, and specifically the retina and posterior eye segment, is often hindered by the physiological protection structures and mechanisms of the organ, as well as by the unsuitable physico-chemical features of the active molecules. Intravitreal injection of drugs and monoclonal antibodies is at present the most common therapeutic procedure to reach the retinal area; however, it is associated with a high risk of side effects and requires the intervention of a physician. One of the ‘dream goals’ in this field is to reach the retinal area using a simple topically applied eye-drop formulation. Research in recent years has progressively made new strategies and technologies available in order to overcome the problems that hinder an efficacious ocular drug bioavailability, leading to even more safe, easy-to-use, and highly compliant therapeutic means. Controlled release as well as nanomedicine approaches, mainly based on polymeric or lipid matrices, are among the most largely explored strategies to pursue this aim. This Topic is aimed at collecting the most recent studies from worldwide laboratories to make an update of the state of the art and open new perspectives toward innovative and effective ocular therapies. In particular, studies dealing with biotech products and gene material will be welcomed, since the association of new therapeutic means with personalized treatments is set to become the most exciting objective for the future of ophthalmology.

We are extremely delighted to present the latest research, and review works that demonstrate the new challenges to achieving an effective ocular drug delivery to increase bioavailability and therapeutic efficacy, while at the same time reducing the risk of side effects. The articles selected for this Topic include the following:

1. “Suprachoroidal Injection: A Novel Approach for Targeted Drug Delivery”.
2. “Innovative Strategies for Drug Delivery to the Ocular Posterior Segment”.
3. “Honey-Related Treatment Strategies in Dry Eye Disease”.
4. “Rutin/Sulfobutylether-β-Cyclodextrin as a Promising Therapeutic Formulation for Ocular Infection”.
5. “Formulating Resveratrol and Melatonin Self-Nanoemulsifying Drug Delivery Systems (SNEDDS) for Ocular Administration Using Design of Experiments”.
6. “Fabrication and Characterization of an Enzyme-Triggered, Therapeutic-Releasing Hydrogel Bandage Contact Lens Material”.
7. “Celecoxib/Cyclodextrin Eye Drop Microsuspensions: Evaluation of In Vitro Cytotoxicity and Anti-VEGF Efficacy for Retinal Diseases”.
8. “A Rapid Screening Platform for Simultaneous Evaluation of Biodegradation and Therapeutic Release of an Ocular Hydrogel”.
9. “Development of ARPE-19-Equipped Ocular Cell Model for In Vitro Investigation on Ophthalmic Formulations”.
10. “Development and Bioactivity of Zinc Sulfate Cross-Linked Polysaccharide Delivery System of Dexamethasone Phosphate”.
11. “Development of Osthole-Loaded Microemulsions as a Prospective Ocular Delivery System for the Treatment of Corneal Neovascularization: In Vitro and In Vivo Assessments”.
12. “Discovery and Potential Utility of a Novel Non-Invasive Ocular Delivery Platform”.
13. “Travoprost Liquid Nanocrystals: An Innovative Armamentarium for Effective Glaucoma Therapy”.

In this way, readers will have the opportunity to read three excellent review articles on different topics, including the use of suprachoroidal injection for drug delivery. The second article covers different innovative strategies for drug delivery to the ocular posterior segment, namely, the use of nanomedicine, liposomes, nanomicelles, dendrimers, organic nanopolymers, ocular inserts, hydrogels, and contact lenses, among others. Finally, the last article covers the use of honey in the treatment of dry eye disease.

As far as the research articles are concerned, several examples of targeted and controlled release of ocular drugs are presented, namely, rutin/sulfobutylether-β-cyclodextrin for ocular injection, resveratrol and melatonin self-nanoemulsifying drug delivery systems (SNEDDS) for ocular administration, enzyme-triggered, therapeutic-releasing hydrogel bandage contact lens material, celecoxib/cyclodextrin eye drop microsuspensions, zinc sulfate cross-linked polysaccharide delivery system of dexamethasone phosphate, osthole-loaded microemulsions for the treatment of corneal neovascularization, and finally, travoprost liquid nanocrystals for the treatment of glaucoma.

We would like to take this opportunity to thank all the authors who have made an exemplary contribution with their valuable literature reviews and scientific research in the field of the ocular drug delivery.

We also believe that the works carried out by everyone and presented in this excellent Topic are exceptional contributions to the continued and future scientific progress in the field of ophthalmic modified drug release.

